# Sugar-sweetened beverages, low/no-calorie beverages, fruit juice and non-alcoholic fatty liver disease defined by fatty liver index: the SWEET project

**DOI:** 10.1038/s41387-023-00237-3

**Published:** 2023-04-21

**Authors:** Novita D. Naomi, Joy Ngo, Elske M. Brouwer-Brolsma, Marion E. C. Buso, Sabita S. Soedamah-Muthu, Carmen Pérez-Rodrigo, Joanne A. Harrold, Jason C. G. Halford, Anne Raben, Johanna M. Geleijnse, Lluis Serra-Majem, Edith J. M. Feskens

**Affiliations:** 1 Division of Human Nutrition and Health, Wageningen University and Research, Wageningen, the Netherlands; 2 Nutrition Research Foundation, Barcelona Science Park, Barcelona, Spain; 3 Center of Research on Psychological Disorders and Somatic Diseases (CORPS) Department of Medical and Clinical Psychology, Tilburg University, Tilburg, the Netherlands; 4 Institute for Food, Nutrition and Health, University of Reading, Berkshire, UK; 5 Department of Psychology, University of Liverpool, Liverpool, UK; 6 School of Psychology, University of Leeds, Leeds, UK; 7 Department of Nutrition, Exercise, and Sports, Faculty of Science, University of Copenhagen, Copenhagen, Denmark; 8 Clinical Research, Copenhagen University Hospital – Steno Diabetes Center Copenhagen, Herlev, Denmark; 9 Research Institute of Biomedical and Health Sciences (IUIBS), University of Las Palmas de Gran Canaria, Preventive Medicine Service, Centro Hospitalario Universitario Insular Materno Infantil (CHUIMI), Canarian Health Service, Las Palmas, Spain; 10 Centro de Investigación Biomédica en Red Fisiopatologia de la Obesidad y la Nutrición (CIBEROBN), Institute of Health Carlos III, Madrid, Spain

**Keywords:** Metabolic syndrome, Diabetes

## Abstract

**Background:**

Sweetened beverage intake may play a role in non-alcoholic fatty liver disease (NAFLD) development, but scientific evidence on their role is limited. This study examined associations between sugar-sweetened beverages (SSB), low/no-calorie beverages (LNCB) and fruit juice (FJ) intakes and NAFLD in four European studies.

**Methods:**

Data for 42,024 participants of Lifelines Cohort, NQPlus, PREDIMED-Plus and Alpha Omega Cohort were cross-sectionally analysed. NAFLD was assessed using Fatty Liver Index (FLI) (≥60). Restricted cubic spline analyses were used to visualize dose–response associations in Lifelines Cohort. Cox proportional hazard regression analyses with robust variance were performed for associations in individual cohorts; data were pooled using random effects meta-analysis. Models were adjusted for demographic, lifestyle, and other dietary factors.

**Results:**

Each additional serving of SSB per day was associated with a 7% higher FLI-defined NAFLD prevalence (95%CI 1.03–1.11). For LNCB, restricted cubic spline analysis showed a nonlinear association with FLI-defined NAFLD, with the association getting stronger when consuming ≤1 serving/day and levelling off at higher intake levels. Pooled Cox analysis showed that intake of >2 LNCB servings/week was positively associated with FLI-defined NAFLD (PR 1.38, 95% CI 1.15–1.61; reference: non-consumers). An inverse association was observed for FJ intake of ≤2 servings/week (PR 0.92, 95% CI: 0.88–0.97; reference: non-consumers), but not at higher intake levels. Theoretical replacement of SSB with FJ showed no significant association with FLI-defined NAFLD prevalence (PR 0.97, 95% CI 0.95–1.00), whereas an adverse association was observed when SSB was replaced with LNCB (PR 1.12, 95% CI 1.03–1.21).

**Conclusions:**

Pooling results of this study showed that SSB and LNCB were positively associated with FLI-defined NAFLD prevalence. Theoretical replacement of SSB with LNCB was associated with higher FLI-defined NAFLD prevalence. An inverse association was observed between moderate intake of FJ and FLI-defined NAFLD. Our results should be interpreted with caution as reverse causality cannot be ruled out.

## Introduction

Non-alcoholic fatty liver disease (NAFLD) is a general term that refers to a broad range of liver disorders, including benign macrovesicular hepatic steatosis (>5% of hepatocytes), non-alcoholic steatohepatitis, hepatic fibrosis, and liver cirrhosis, which constitutes the third most common cause of hepatocellular carcinoma [1]. Globally, NAFLD prevalence is estimated to be around 25%, which has steadily increased and currently coincides with obesity, type 2 diabetes, and metabolic syndrome epidemic [2].

Major determinants of NAFLD include sedentary lifestyles and poor-quality diets, namely high sugar intake [3–5]. A recent meta-analysis of 12 studies (nine cross-sectional, two case-control and one cohort study) showed that sugar-sweetened beverages (SSB) intake was associated with a 39% (95% CI 1.29–1.50) higher NAFLD risk [6]. However, studies on the association between SSB intake and NAFLD were mainly conducted in relatively small studies using liver imaging techniques or biopsies to define NAFLD, which are often not feasible in large population studies [6, 7]. Surrogate formulas—employing serum biomarkers—have been developed, validated and widely used in recent decades, including the Fatty Liver Index (FLI) by Bedogni et al. [8–10], to facilitate non-invasive and simple NAFLD assessment in larger studies. However, evidence on the association between SSB and NAFLD detected with FLI in larger studies is still limited [3, 11, 12].

Low/no-calorie sweeteners have been increasingly used as an alternative to sugar. However, evidence on the association between low/no calorie beverages (LNCB) and NAFLD is limited and generally shows no association [7, 13, 14]. Besides LNCB, fruit juice (FJ) is also often considered an alternative to SSB, although some argue that FJ contains high amounts of sugar apart from its vitamin and polyphenol content. Population-based studies showed beneficial associations with cardiometabolic health at moderate doses of FJ consumption [15–17], but evidence specifically on NAFLD is lacking.

Given all the aforementioned considerations, we aimed to investigate the association between SSB, LNCB, and FJ intakes and FLI-defined NAFLD in four European studies. Additionally, we investigated whether LNCB and FJ could be used as an alternative to SSB by means of substitution analyses which is currently lacking in the literature.

## Methods

### Study design and population

The SWEET project is a European Union-funded project that aims to investigate public health and safety, obesity, and sustainability risks and benefits of replacing sugar with sweeteners and sweetness enhancers (www.sweetproject.eu). The results presented in this manuscript are based on harmonized data of the Lifelines Cohort study (Lifelines; The Netherlands), the Nutrition Questionnaire Plus study (NQPlus; The Netherlands), the PREDIMED-Plus study (PREDIMED-Plus; Spain), and the Alpha Omega Cohort (AOC; The Netherlands). All participants in all cohorts gave written informed consent before participating.

### Lifelines

Lifelines is a multidisciplinary prospective population-based cohort study examining in a unique three-generation design the health and health-related behaviours of 167,729 persons living in the North of The Netherlands [18, 19]. It employs a broad range of investigative procedures in assessing biomedical, sociodemographic, behavioural, physical and psychological factors that contribute to the health and disease of the general population, with a special focus on multi-morbidity and complex genetics. Between 2006 and 2013, participants aged 0–93 yr were recruited to undergo baseline measurements. For present analysis, data for 152,728 participants aged ≥18 yr were included. After consecutive exclusion of those with missing dietary data (*n* = 8633), implausible energy intake (<800 or >4000 kcal/day for men or <500 or >3500 kcal/day for women; *n* = 15,483) [20], missing FLI data (*n* = 86,137), history of hepatitis (*n* = 31), excessive alcohol consumption (>20 g/day for women and >30 g/day for men; *n* = 1825), or missing covariates (*n* = 3679), *n* = 36,940 remained for current analyses (Supplemental Table 1). Lifelines has been approved by the Medical Ethical Review Committee of the University Medical Center in Groningen.

### NQPlus

NQPlus is a prospective cohort study involving mainly Caucasian Dutch adults aged 20–70 yr from the central part of the Netherlands (Wageningen, Ede, Renkum, Arnhem, Barneveld, Veenendaal) [21]. NQPlus’ objectives were to establish a database for national dietary assessment reference to produce and validate food frequency questionnaires (FFQs), as well as to provide a database for longitudinal study in dietary factors and health-related outcomes. Extensive data collection was performed on habitual dietary intake and various health outcomes, including cardiometabolic health parameters. Between 2011 and 2013, 2048 participants were included in the study. After participants with missing dietary data (*n* = 401), implausible energy intake (*n* = 20), missing FLI data (*n* = 56), history of hepatitis (*n* = 32) or excessive alcohol consumption (*n* = 184) were consecutively excluded, *n* = 1341 remained (Supplemental Table 1). Due to the high percentages of missing covariates (*n* physical activity = 110 [8%], *n* sedentary behaviour = 111 [8%], *n* smoking status = 126 [9%]), multiple imputations were applied using “mice” package in R and five datasets were produced [22]. NQPlus was approved by the ethical committee of Wageningen University and Research.

### PREDIMED-Plus

PREDIMED-Plus is a multicentre randomized controlled trial which was conducted based on the idea of the previous study, the PREDIMED (in Spanish: PREvención con DIeta MEDiterránea) trial [23]. PREDIMED-Plus aims to examine the effect of a traditional Mediterranean diet with energy restriction and increased physical activity on weight reduction and cardiovascular incidence and mortality. PREDIMED-Plus was conducted in 23 study centres across Spain. From September 2013 to November 2016, PREDIMED-plus recruited men aged 55–75 yr and women aged 60–75 yr with overweight or obesity who met at least three metabolic syndrome criteria according to the International Diabetes Federation and the AHA/National Heart, Lung and Blood Institute [24]. For present analysis, data from the Canary Island study centre were used, which included information for 266 participants with complete dietary data. Participants with implausible energy intake (*n* = 3), missing FLI (*n* = 10), and excessive alcohol consumption (*n* = 1) were consecutively excluded, and *n* = 250 remained for the current analyses (Supplemental Table 1). PREDIMED-Plus was registered at the International Standard Randomized Controlled Trial database (ISRCTN; 89898870) and the protocol was approved by local Research Ethics Committees.

### AOC

AOC is a prospective cohort study of 4837 Dutch patients aged 60–80 yr who had experienced a myocardial infarction (MI) within 10 yr before study enrolment [25, 26]. The cohort is continuously followed for cause-specific mortality. Baseline measurements took place in 2002 and 2006 during which patients filled in questionnaires on demographics, lifestyle, diet, medication use and medical history, and underwent physical examination by trained research nurses. For current analyses, patients with missing dietary data (*n* = 453) or implausible dietary intake (*n* = 24), missing FLI (*n* = 179), excessive alcohol consumption (*n* = 647), or missing covariates (*n* = 41) were excluded, resulting in a total of *n* = 3493 remaining for analysis (Supplemental Table 1). AOC was registered at ClinicalTrials.gov (NCT03192410) and was approved by a central Medical Ethics Committee in the Netherlands.

### Dietary assessment

Dietary intake data were collected using a newly developed and validated 110-item FFQ [27] and 183-item semi-quantitative FFQ [21, 28] in Lifelines and NQPlus, respectively, which covered all major food groups. These FFQs were created based on the Dutch consumption survey and were validated for energy and a range of nutrients as well as intake biomarkers. Average daily nutrient intake was calculated by multiplying consumption intake frequency by portion size and nutrient content in grams as indicated in the Dutch Food Composition Table (NEVO) 2011. In PREDIMED-Plus, dietary intake was collected using a 143-item FFQ [23, 29, 30], based on usual food consumption in Spain and designed to assess overall diet. This FFQ has been validated in different studies using four 3- or 4-day food records. Average daily nutrient intake was calculated by multiplying consumption intake frequency by portion size and nutrient content in the Spanish food composition tables. Within AOC, dietary data were collected using a semi-quantitative 203-item FFQ, a modified and extended version of the validated FFQ that was specifically designed to estimate fatty acids and cholesterol intake [31]. The Dutch Food Composition (NEVO) 2006 was used to convert food intake to energy and nutrient intakes in AOC. All population studies include intake measures of SSB, LNCB, and FJ, except for AOC where LNCB was combined with water, which therefore could not be analysed individually. Definition of SSB and LNCB covers soft drinks or lemonade sweetened with sugar or sugar substitutes. Coffee or tea sweetened with sugar or sweeteners as well as sweetened dairy drinks were not included in the definition of SSB and LNCB. FJ was defined as fruit juice i.e., apple juice, orange juice and mixed fruit juice, which were mainly pasteurized. SSB, LNCB, and FJ intakes were presented as servings of 150 ml.

### Assessment and definition of NAFLD

Blood samples were collected after an overnight fast in all studies, except for AOC where overnight fasting blood samples were collected in 36% of all participants. Blood lipids including total cholesterol and triglycerides (TG) as well as liver enzymes including gamma-glutamyl transferase (GGT) were measured using routine procedures on a Roche Modular P800 chemistry analyser in Lifelines, Dimension Vista 1500 automated analyser or a Roche Modular P800 chemistry analyser in NQplus, and Cobas 8000 c 702 Module Roche Diagnostics in PREDIMED-Plus. In AOC, TG was analysed using Roche Hitachi 912, whereas liver enzymes were determined using automated analyses Abbott Architect ci8200. Anthropometry measurements (weight, height and waist circumference) were performed by well-trained staff. Body weight and height were measured without shoes. Body mass index (BMI) was calculated as weight divided by squared height (kg/m^2^). NAFLD was defined using FLI ≥ 60, which has been established by Bedogni et al. (2006) [8]. FLI is calculated based on serum concentrations of TG and GGT, BMI, and waist circumference, applying the following formula:

FLI = (*e*^L^/1 + *e*^L^)×100, where *L* = 0.953 × log(TG) + 0.139 × BMI + 0.718 × log(GGT) + 0.053 × waist circumference−15.745.

### Assessment of covariates

Information on sociodemographic, lifestyle, and disease history was obtained from self- or interviewer-administered questionnaires. Educational levels was categorized into low (less than secondary qualification), medium (from secondary qualification up to university with no bachelor’s degree) or high (Bachelor’s, Master’s, or Ph.D. degree). Smoking status was categorized as non-smoker, former smoker, or current smoker. In Lifelines and NQplus, physical activity and sedentary behaviours (TV-watching) was assessed using the Short Questionnaire to Assess Health (SQUASH) [32] and the Activity Questionnaire for Adults and Adolescents (AQuAA) [33], respectively. In PREDIMED-Plus, physical activity was assessed using the Physical Activity Readiness Questionnaire (PAR-Q) as well as the Rapid Assessment of Physical Activity questionnaires 1 and 2 (RAPA-1 and RAPA-2) [34]. Sedentary activity in PREDIMED-Plus was assessed by using the Nurses’ Health Study physical activity questionnaire validated for the Spanish population [35]. Physical activity in Lifelines, NQPlus and PREDIMED-Plus was reported in Metabolic equivalent (MET)-min/week for moderate-level activity and in min/week for sedentary behaviour. In AOC, physical activity was assessed by using validated the Physical Activity Scale for the Elderly [36], and categorized as low (no activity/light activity, ≤3 METs), moderate (0–5 day/week moderately or vigorously active, >3 METs) or high (≥5 day/week moderately or vigorously active, >3 METs) physical activity. Alcohol intake was assessed using FFQ from which ethanol intake was calculated and categorized as 0, >0–≤10, >10–≤20, or >20 g/day.

### Statistical analysis

Baseline characteristics were presented as mean with standard deviation for normally distributed continuous variables or as a median and interquartile range for variables with skewed distribution. Categorical variables were shown as numbers and percentages. Dose–response associations between SSB, LNCB, and FJ intakes and FLI-defined NAFLD prevalence were first analysed using restricted cubic spline analyses (3 knots) [37]. To ensure adequate power and precision, restricted cubic spline analyses were only performed for Lifelines. The fit of the spline model was examined against a linear model with the likelihood-ratio test. Cox proportional hazard regression with robust variance estimate was used to investigate the associations of SSB, LNCB and FJ intakes with FLI-defined NAFLD prevalence with a 95% confidence interval (PR [95% CI]) in each cohort, whereas theoretical substitution analyses were conducted using the leave-one-out model, where the model included SSB, LNCB, and FJ (in servings/day) as one variable followed by beverage defined as a replacement. Results of individual cohorts were subsequently pooled using random effect meta-analyses. To explore the potential of reverse causality, sensitivity analyses were conducted in Lifelines after excluding those with self-reported medical conditions (i.e., diabetes, CVD, hypercholesterolemia, or hypertension). All analyses were adjusted for age, sex (Model 1), educational levels (low, medium, or high), lifestyle factors including moderate physical activity (MET-min/week or in categories as in AOC), sedentary behaviour (min/week), smoking status (never, former, or current smoker), and alcohol use (0, >0–≤10, >10–≤20, or >20 g/day) (Model 2), intakes of other food groups including grains (g/day), potatoes (g/day), vegetables (g/day), fruit (g/day), meat and processed meat (g/day), dairy (g/d), coffee (ml/day), tea (ml/day), legumes (g/day), nuts (g/day), oils and fats (g/day), sugary foods (candy, sweet snack i.e., cookies, milk chocolate, any kind of toppings and popsicle) (g/day), and mutual adjustment for other beverages (SSB, LNCB, and FJ in g/day) as well as total energy intake (kcal/d) (Model 3). Statistical analysis was performed using R 4.0.2 and RStudio 1.3.959 for NQplus, PREDIMED-Plus, and AOC, and RStudio 2022.02.0 for Lifelines.

## Results

### General characteristics of participants

The mean (SD) age ranged from 45 (7) yr in Lifelines to 69 (6) yr in AOC (Table 1). More than half of the participants in Lifelines (61%), NQPlus (52%) and PREDIMED-Plus (66%) were women, whereas the opposite was observed in AOC (women 23%). Most participants reported low/moderate education, except for NQPlus participants of whom 54% reported higher education levels. Most participants never smoked, except for AOC participants of whom 66% were former smokers. Median (IQR) SSB intake ranged from 0.0 (0.0–0.1) serving/day in NQPlus and PREDIMED-Plus, to 0.1 (0.0–0.6) serving/day in Lifelines. Similar patterns were observed for LNCB. Median (IQR) FJ intake ranged from 0.1 (0.0–0.6) serving/day in NQPlus to 0.4 (0.0–1.1) serving/day in PREDIMED-Plus. FLI-defined NAFLD prevalence was 22% in Lifelines and NQplus, 60% in AOC and 78% in PREDIMED-Plus (Table 2).

**Table 1:** General characteristics of Lifelines, NQPlus, PREDIMED-Plus and AOC participants.

Characteristics^a^	Lifelines	NQPlus^b^	PREDIMED-Plus	AOC
*n*	36,940	1341	250	3493
Age, *yr*	45 ± 13	52 ± 12	65 ± 4	69 ± 6
Women	22,504 (61)	657 (49)	166 (66)	786 (23)
Education				
Low	1618 (4)	9 (1)	175 (70)	2037 (58)
Moderate	23,978 (65)	608 (45)	64 (26)	1074 (31)
High	11,344 (31)	724 (54)	11 (4)	382 (11)
Smoking status				
Never	17,053 (46)	668 (55)	171 (68)	631 (18)
Former	12,401 (34)	461 (38)	69 (28)	2300 (66)
Current	7476 (20)	86 (7)	10 (4)	562 (16)
Moderate physical activity, *MET-min/week*	1680 [818, 2988]	798 [210, 1680]	350 [0, 1399]	731 (21)^c^
Sedentary behaviour, *min/week*	840 [630, 1260]	1800 [1200, 2700]	1842 ± 789	NA
Alcohol use				
0 g/day	1008 (3)	76 (6)	109 (44)	211 (6)
>0–≤10 g/day	27,177 (74)	845 (63)	109 (44)	2202 (63)
>10–≤20 g/day	7519 (20)	306 (23)	28 (11)	766 (22)
>20 g/day	1236 (3)	114 (9)	4 (2)	314 (9)
Prevalent diabetes	882 (2)	45 (3)	79 (32)	592 (17)
Hypertension	7948 (22)	329 (25)	225 (90)	1719 (49)
History of CVD	890 (2)	40 (3)	All	All
*Metabolic markers*				
FLI	26.9 [11.1, 55.7]	27.0 [10.6, 55.9]	78.6 [63.7, 88.8]	67.3 [47.8, 83.5]
TG, *mg/dL*	104 ± 69	91 [67, 129]	132 [97, 169]	145 [106, 204]
GGT, *U/L*	20.0 [15.0–29.0]	18.3 [13.3, 27.0]	22.0 [16.0, 30.0]	32.0 [24.0, 46.0]
Waist circumference, *cm*	90.3 ± 12.3	91.2 ± 12.7	107.0 ± 9.9	101.7 ± 10.5
BMI, *kg/m*^*2*^	26.0 ± 4.3	25.9 ± 4.1	32.5 ± 3.5	27.7 ± 3.8
BMI ≥25	20,373 (55)	721 (54)	250 (100)	2687 (77)
ALT	19.0 [14.0, 27.0]	24.1 [18.9, 30.7]	18.0 [13.8, 24.0]	16.0 [13.0, 21.0]
AST	23.0 [19.0–27.00]	21.4 [18.0, 27.0]	18.0 [16.8, 24.0]	27.0 [24.0, 32.0]
*Dietary intakes*				
SSB, *servings/day*	0.1 [0.0, 0.6]	0.0 [0.0, 0.1]	0.0 [0.0, 0.1]	0.1 [0.0, 0.5]
LNCB, *servings/day*	0.1 [0.0, 0.6]	0.0 [0.0, 0.1]	0.0 [0.0, 0.0]^d^	NA
FJ, *servings/day*	0.2 [0.0, 0.7]	0.1 [0.0, 0.6]	0.4 [0.0, 1.1]	0.4 [0.1, 1.0]
Total energy, *kcal/day*	2028 ± 566	2013 ± 555	2071 ± 532	1875 ± 509
Grains, *g/day*	191 ± 80	195 ± 87	122 ± 57	169 ± 59
Potatoes, *g/day*	92 ± 54	67 [37,97]	50 [39,96]	94 ± 45
Vegetables, *g/day*	103 ± 57	156 ± 86	269 ± 110	71 [52,94]
Fruits, *g/day*	110 [42, 220]	210 [83, 238]	404 ± 179	116 [54, 262]
Meat and processed meat, *g/day*	77 ± 36	70 ± 41	101 ± 50	77 [44, 107]
Dairy, *g/day*	270 [167, 401]	299 ± 182	460 ± 244	245 [162, 380]
Coffee, *ml/day*	424 ± 277	406 [174, 638]	50 [50, 125]	375 [375, 562]
Tea, *ml/day*	232 [45, 348]	174 [67, 406]	0 [0, 3]	150 [45, 450]
Nuts, *g/day*	7 [3,16]	12 [6,21]	13 [4,30]	3 [1,7]
Legumes, *g/day*	11 [0, 28]	37 [19,75]	59 ± 29	21 [12,37]
Oils and fats, *g/day*	22 [12,32]	27 ± 17	21 ± 10	37 ± 3
Sugary food, g/day	63 [39,95]	51 ± 35	46 [24,80]	64 [34, 101]

*AOC* Alpha Omega Cohort, *ALT* alanine transaminase, *AST* aspartate aminotransferase, *CVD* cardiovascular disease, *FJ* fruit juice, *FLI* Fatty Liver Index, *GGT* gamma-glutamyl transferase, *LNCB* low/no-calorie sweetened beverages, *MET* metabolic equivalent task, *NQPlus* Nutrition Questionnaire Plus, *SSB* sugar-sweetened beverages, *TG* triglycerides.

^a^Value are mean ± SD, median [25th, 75th percentile], or *n* (%) as indicated.

^b^*n* physical activity = 1231, *n* sedentary activity = 1230, *n* smoking status = 1251.

^c^In categories: no/light activity *n* = 1465 (42%), moderate physical activity is presented in the table, high physical activity *n* = 1297 (37%).

^d^Intake ranged from 0.00 to 3.33 serving/day.

**Table 2:** Associations of SSB, LNCB, and FJ intakes with FLI-defined NAFLD prevalence for each serving per day increment in all cohorts.

	PR (95% CI)					
	Lifelines	NQPlus^a^	PREDIMED-Plus	AOC	Pooled	
*n* total	36,940	1341	250	3493	PR (95% CI)	*I*^2^, *p-*value
*n* (%) cases	8200 (22)	290 (22)	195 (78)	2092 (60)		
*SSB*						
Model 1^b^	1.11 (1.09–1.13)	1.02 (0.82–1.26)	1.01 (0.79–1.29)	1.05 (1.03–1.07)	1.07 (1.02–1.13)	86%, <0.01
Model 2^c^	1.06 (1.04–1.08)	0.99 (0.81–1.22)	1.01 (0.77–1.31)	1.04 (1.02–1.07)	1.05 (1.04–1.07)	0%, 0.55
Model 3^d^	1.09 (1.07–1.11)	0.94 (0.76–1.17)	1.01 (0.74–1.37)	1.05 (1.02–1.08)	1.07 (1.03–1.11)	58%, 0.07
*LNCB*						
Model 1^b^	1.24 (1.22–1.26)	1.35 (1.19–1.53)	1.08 (0.93–1.25)	NA	1.23 (1.12–1.34)	73%, 0.02
Model 2^c^	1.21 (1.19–1.23)	1.29 (1.14–1.45)	1.08 (0.94–1.25)	NA	1.20 (1.12–1.29)	58%, 0.09
Model 3^d^	1.20 (1.18–1.22)	1.22 (1.08–1.37)	1.12 (0.96–1.31)	NA	1.20 (1.18–1.22)	0%, 0.57
*FJ*						
Model 1^b^	1.05 (1.02–1.08)	0.99 (0.81–1.20)	1.03 (0.96–1.11)	0.99 (0.96–1.02)	1.02 (0.98–1.06)	63%, 0.04
Model 2^c^	1.05 (1.02–1.08)	0.96 (0.80–1.16)	1.03 (0.96–1.10)	1.00 (0.97–1.03)	1.02 (0.99–1.06)	51%, 0.11
Model 3^d^	1.07 (1.04–1.10)	0.96 (0.81–1.14)	1.03 (0.94–1.12)	1.01 (0.98–1.04)	1.03 (0.99–1.08)	67%, 0.03

*AOC* Alpha Omega Cohort, *CI* confidence interval, *FJ* fruit juice, *LNCB* low/no-calorie sweetened beverages, *NQPlus* Nutrition Questionnaire Plus, *PR* prevalence ratio, *SSB* sugar-sweetened beverages.

^a^Imputed with multiple imputation method.

^b^Model 1: adjusted for age and sex.

^c^Model 2: model 1 with additional adjustment for education levels, moderate physical activity, sedentary behaviour, smoking status, alcohol intake.

^d^Model 3: model 2 with additional adjustment for grains, potatoes, vegetable, fruit, meat and processed meat, dairy, coffee, tea, legumes, nuts, oils and fats, sugary foods, mutual adjustment for other beverages (SSB, LNCB, or FJ, except for AOC where LNCB consumption data was not available), and energy intake.

In most cohorts, participants with a higher SSB intake were more likely to be younger, men and less physically active. The opposite trends were observed in AOC. In all cohorts, SSB consumers were more likely to be diagnosed with diabetes, had a higher intake of meat, sugary food and total energy, and lower intake of vegetables and fruit (Supplemental Table 2). Participants with higher LNCB intake were more likely to be younger, less physically active, to be diagnosed with diabetes, hypercholesterolemia or hypertension, have a BMI ≥25, higher meat intake, and lower intake of vegetables and fruit (Supplemental Table 3). For FJ, participants with a higher intake were more likely to have BMI <25 and higher sugary food consumption (Supplemental Table 4).

### Association between SSB, LNCB and FJ intakes and FLI-defined NAFLD prevalence

**Fig. 1:**
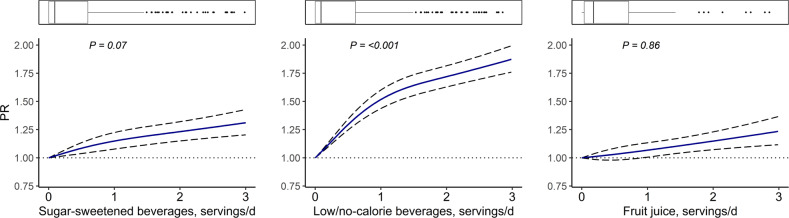
Dose–response associations of SSB, LNCB and FJ intakes with FLI-defined NAFLD in Lifelines. Three knots with 0 g/day as a reference value were placed and model was adjusted for age, sex, education levels, moderate physical activity, sedentary behaviour, smoking status, alcohol intake, grains, potatoes, vegetable, fruit, meat and processed meat, dairy, coffee, tea, legumes, nuts, oils and fats, sugary foods, mutual adjustment for other beverages (SSB, LNCB, or FJ), and energy intake. CI confidence interval, FJ fruit juices, LNCB low/no-calorie sweetened beverages, PR prevalence ratio, SSB sugar-sweetened beverages.

Dose–response analysis in Lifelines did not provide strong evidence of a non-linear association between SSB and FLI-defined NAFLD (*P* non-linearity = 0.07) (Fig. 1). Pooled results of four studies showed an association between each additional serving/day of SSB and higher prevalence of FLI-defined NAFLD (PR 1.07, 95% CI 1.03–1.11) (Table 2). Although there was evidence of interaction between SSB and age categories, results from a stratified analysis in Lifelines by age as well as by sex did not indicate significant differences (Supplemental Table 6). Sensitivity analysis by excluding those with self-reported disease in Lifelines did not alter the associations (Supplemental Table 7).

Dose–response analysis in Lifelines showed strong evidence of a non-linear association between LNCB and FLI-defined NAFLD prevalence (*P* non-linearity = <0.001) (Fig. 1), with the association getting stronger when consuming ≤1 serving/day and levelling off at higher intake levels. Subsequent Cox regression showed that, compared to no intake, LNCB intake of ≤2 servings/week was associated with a PR of 1.13 (95% CI 1.00–1.26), whereas intake of >2 servings/week was associated with a PR of 1.38 (95% CI 1.15–1.61) (Table 3). In Lifelines, stratified analysis by sex showed a stronger association in women (PR 1.65, 95% CI 1.54–1.77) than in men (PR 1.36, 95% CI 1.29–1.43) when comparing those with LNCB intake >2 servings/week with non-consumers (Supplemental Table 6). Excluding participants with self-reported disease did not alter the main findings (Supplemental Table 7). Theoretical replacement of SSB with the same amount of LNCB was associated with a higher prevalence of FLI-defined NAFLD (PR 1.12, 95% CI 1.03–1.21) (Table 4).

**Table 3:** Associations of SSB, LNCB, and FJ intakes with FLI-defined NAFLD prevalence for categories of beverage consumption in all cohorts.

	PR (95% CI)								
	SSB			LNCB			FJ		
	No intake	≤2 servings/week	>2 servings/week	No intake	≤2 servings/week	>2 servings/week	No intake	≤2 servings/week	>2 servings/week
Lifelines									
*N* total	13949	9262	14,122	14,476	7929	14,305	8154	11,747	17,039
*N*(%) cases	3325 (24)	1438 (18)	3781 (27)	2897 (20)	1454 (18)	3849 (27)	2180 (27)	2322 (20)	3698 (22)
Model 1^b^	1 (ref)	0.75 (0.71–0.79)	1.08 (0.99–1.09)	1 (ref)	0.95 (0.90–1.01)	1.51 (1.45–1.58)	1 (ref)	0.78 (0.75–0.82)	0.87 (0.83–0.90)
Model 2^c^	1 (ref)	0.77 (0.73–1.04)	0.99 (0.95–1.04)	1 (ref)	1.00 (0.95–1.06)	1.50 (1.44–1.56)	1 (ref)	0.85 (0.81–0.90)	0.94 (0.90–0.98)
Model 3^d^	1 (ref)	0.90 (0.86–0.95)	1.08 (1.03–1.13)	1 (ref)	1.05 (0.99–1.11)	1.48 (1.42–1.54)	1 (ref)	0.92 (0.87–0.96)	1.01 (0.97–1.06)
*NQPlus*^a^									
*N* total	737	412	192	883	268	190	292	493	556
*N*(%) cases	155 (21)	90 (22)	45 (23)	160 (18)	65 (24)	65 (34)	66 (23)	109 (22)	115 (21)
Model 1^b^	1 (ref)	0.95 (0.76–1.20)	1.03 (0.76–1.39)	1 (ref)	1.28 (1.00–1.64)	1.78 (1.40–2.26)	1 (ref)	0.97 (0.74–1.26)	0.91 (0.70–1.18)
Model 2^c^	1 (ref)	0.97 (0.77–1.22)	1.01 (0.74–1.36)	1 (ref)	1.22 (0.96–1.57)	1.67 (1.30–2.13)	1 (ref)	0.99 (0.76–1.28)	0.93 (0.71–1.20)
Model 3^d^	1 (ref)	1.06 (0.84–1.34)	1.02 (0.74–1.39)	1 (ref)	1.17 (0.91–1.51)	1.50 (1.18–1.91)	1 (ref)	1.08 (0.83–1.41)	1.02 (0.79–1.32)
*PREDIMED-Plus*									
*N* total	180	49	21	189	45	16	77	41	132
*N*(%) cases	139 (77)	10 (80)	17 (81)	142 (75)	40 (89)	13 (81)	58 (75)	28 (68)	109 (83)
Model 1^b^	1 (ref)	1.02 (0.87–1.21)	1.00 (0.79–1.26)	1 (ref)	1.20 (1.04–1.37)	1.08 (0.85–1.37)	1 (ref)	0.92 (0.72–1.18)	1.10 (0.95–1.28)
Model 2^c^	1 (ref)	1.00 (0.85–1.17)	0.97 (0.78–1.22)	1 (ref)	1.22 (1.07–1.40)	1.07 (0.85–1.34)	1 (ref)	0.92 (0.73–1.16)	1.10 (0.94–1.28)
Model 3^d^	1 (ref)	0.97 (0.83–1.14)	0.96 (0.75–1.24)	1 (ref)	1.23 (1.07–1.43)	1.12 (0.89–1.42)	1 (ref)	0.95 (0.76–1.20)	1.13 (0.96–1.32)
*AOC*									
*N* total	1577	721	1195	NA	NA	NA	873	630	1990
*N*(%) cases	903 (57)	426 (59)	761 (64)				567 (65)	362 (58)	1163 (58)
Model 1^b^	1 (ref)	1.02 (0.94–1.10)	1.09 (1.02–1.15)	NA	NA	NA	1 (ref)	0.89 (0.82–0.96)	0.91 (0.85–0.96)
Model 2^c^	1 (ref)	1.03 (0.95–1.10)	1.08 (1.02–1.15)	NA	NA	NA	1 (ref)	0.90 (0.83–0.98)	0.94 (0.89–1.00)
Model 3^d^	1 (ref)	1.03 (0.96–1.11)	1.09 (1.02–1.16)	NA	NA	NA	1 (ref)	0.92 (0.85–1.00)	0.97 (0.91–1.03)
*Pooled*									
Model 3^d^		0.97 (0.89–1.05)	1.08 (1.04–1.12)		1.13 (1.00–1.26)	1.38 (1.15–1.61)		0.92 (0.88–0.97)	1.00 (0.97–1.04)
*I*^2^, *p* value		75%, <0.01	0%, 0.78		72%, 0.03	78%, 0.01		0%, 0.74	18%, 0.30

*AOC* Alpha Omega Cohort, *CI* confidence interval, *FJ* fruit juice, *LNCB* low/no-calorie sweetened beverages, *NQPlus* Nutrition Questionnaire Plus, *PR* prevalence ratio, *SSB* sugar-sweetened beverages.

^a^Imputed with multiple imputation method.

^b^Model 1: adjusted for age and sex.

^c^Model 2: model 1 with additional adjustment for education levels, moderate physical activity, sedentary behaviour, smoking status, alcohol intake.

^d^Model 3: model 2 with additional adjustment for grains, potatoes, vegetable, fruit, meat and processed meat, dairy, coffee, tea, legumes, nuts, oils and fats, sugary foods, mutual adjustment for other beverages (SSB, LNCB, or FJ, except for AOC where LNCB consumption data was not available), and energy intake.

**Table 4:** Adjusted associations of SSB, LNCB, and FJ consumption and FLI-defined NAFLD prevalence when replacing SSB with the same amount of LNCB or FJ.

	PR (95%CI)					
	Lifelines	NQPlus^a^	PREDIMED-Plus	AOC	Pooled	
*n* total	36,940	1341	250	3493	PR	*I*^2^, *p-*value
*n* (%) cases	8200 (22)	290 (22)	195 (78)	2092 (60)		
*SSB by LNCB*						
Model 1^b^	1.11 (1.08–1.14)	1.36 (1.06–1.73)	1.08 (0.82–1.42)	NA	1.16 (1.02–1.30)	50%, 0.13
Model 2^c^	1.13 (1.10–1.15)	1.32 (1.04–1.69)	1.08 (0.80–1.44)	NA	1.15 (1.06–1.23)	19%, 0.29
Model 3^d^	1.13 (1.11–1.16)	1.30 (1.02–1.65)	1.10 (0.81–1.51)	NA	1.14 (1.09–1.18)	0%, 0.38
Model 4^e^	1.10 (1.07–1.13)	1.28 (1.00–1.63)	1.11 (0.80–1.53)	NA	1.12 (1.03–1.21)	3%, 0.36
*SSB by FJ*						
Model 1^b^	0.92 (0.88–0.95)	0.97 (0.72–1.29)	1.03 (0.78–1.35)	0.94 (0.91–0.98)	0.93 (0.91–0.96)	0%, 0.77
Model 2^c^	0.97 (0.93–1.00)	0.96 (0.71–1.29)	1.02 (0.76–1.36)	0.96 (0.92–0.99)	0.97 (0.94–0.99)	0%, 0.96
Model 3^d^	0.99 (0.96–1.03)	1.02 (0.76–1.38)	1.01 (0.74–1.39)	0.96 (0.93–1.00)	0.98 (0.95–1.00)	0%, 0.69
Model 4^e^	0.98 (0.95–1.02)	1.00 (0.74–1.36)	1.02 (0.74–1.39)	0.96 (0.93–1.00)	0.97 (0.95–1.00)	0%, 0.87

*AOC* Alpha Omega Cohort, *CI* confidence interval, *FJ* fruit juice, *LNCB* low/no-calorie sweetened beverages, *NQPlus* Nutrition Questionnaire Plus, *PR* prevalence ratio, *SSB* sugar-sweetened beverages.

^a^Imputed with multiple imputation method.

^b^Model 1: adjusted for age and sex.

^c^Model 2: model 1 with additional adjustment for education levels, moderate physical activity, sedentary behaviour, smoking status, alcohol intake.

^d^Model 3: model 2 with additional adjustment for grains, potatoes, vegetable, fruit, meat and processed meat, dairy, coffee, tea, legumes, nuts, oils and fats, sugary foods, and mutual adjustment for other beverages (SSB, LNCB, or FJ, except for AOC where LNCB consumption data was not available).

^e^Model 4: model 3 with additional adjustment for energy intake.

Dose–response analysis in Lifelines did not suggest evidence of a nonlinear association between FJ and FLI-defined NAFLD prevalence (*P* non-linearity = 0.86) (Fig. 1). However, pooled analysis by categories showed an inverse association between FJ and FLI-defined NAFLD at intake levels of ≤2 servings/week (PR 0.92, 95% CI 0.88–0.97), but no association was seen at higher intake levels (PR 1.00, 95% CI 0.97–1.04) when compared to no intake (Table 3). After excluding those with self-reported disease, the associations did not change significantly (Supplemental Table 6). Finally, there was no evidence for an association between theoretical replacement of SSB with the same amount of FJ and FLI-defined NAFLD (PR 0.97, 95% CI: 0.95–1.00) (Table 4).

## Discussion

Harmonized data analyses of four European studies encompassing both general population and cardiometabolic subjects showed positive associations between SSB and LNCB intakes and FLI-defined NAFLD prevalence. Dose-response analyses in Lifelines showed a non-linear positive association between LNCB and FLI-defined NAFLD with the stronger association at intake levels of <1 serving/day and levelling off at higher intake levels. A beneficial association was observed for moderate FJ intake ranging from ≤2 servings/day when compared to no intake. Theoretical replacement of SSB with the same amount of LNCB showed a positive association with FLI-defined NAFLD, whereas no significant association was observed when replacing SSB with FJ.

Overall, our findings on the association between SSB and FLI-defined NAFLD prevalence are consistent with previous research [6, 7, 11, 38, 39]. A meta-analysis of 12 studies (nine cross-sectional, two case-control and one cohort study) showed a 39% (95% CI 1.29–1.50) higher NAFLD risk—as assessed using liver imaging or biopsy—when comparing highest SSB intake group with non-consumer [6]. Furthermore, a recent study by Zhang et al. in 14,845 participants showed a 47% (HR 95% CI 1.25–1.23) and 59% (HR 95% CI 1.07–2.37) higher prevalence of NAFLD when NAFLD was assessed using abdominal ultrasonography and serum biomarkers (hepatic steatosis index) [11]. In addition, results from short-term trials, experimental studies, and meta-analyses of RCT evaluating individual metabolic markers further confirm our findings [40–44]. The biological explanation of the adverse association between SSB intake and NAFLD can be explained by several biological mechanisms. Its high fructose content may induce hepatic de novo lipogenesis and possibly insulin resistance [45, 46]. Moreover, lack of energy compensation after liquid calorie intake affects energy balance disruption, which contributes to weight gain, an important factor in NAFLD development [47].

We observed positive associations between LNCB, as well as between theoretical replacement of SSB with LNCB and FLI-defined NAFLD prevalence. To date, evidence on LNCB and NAFLD is still limited [7, 13, 14], especially from large studies. In line with our findings, prospective analysis among 1636 women participating in the Framingham Heart Study showed a 48% higher risk of NAFLD and LNCB—when NAFLD was detected using computed liver attenuation measurements [7]. However, this association disappeared after further adjustment for BMI (OR 1.11, 95% CI 0.80–1.56). A prospective study in women with a history of gestational diabetes also showed no association between LNCB and the majority of NAFLD markers when the models were adjusted by various covariates, particularly pre-pregnancy BMI, as well as when performing sensitivity analyses excluding those with chronic diseases - indicating that reverse causality may be the cause [14]. Within our study, participants at higher intake levels of LNCB tended to have a BMI ≥ 25, which might also indicate the presence of reverse causality. Participants with NAFLD or elevated NAFLD parameters may have switched from SSB to LNCB to control their health, e.g., weight regulation. Evidence from experimental studies was summarized in a meta-analysis of 12 RCTs involving 601 adults with overweight or obesity showing that replacing SSB with LNCB reduced body weight, body fat, BMI, intrahepatocellular lipid, but not other clinical parameters including liver enzymes i.e. alanine transaminase and aspartate aminotransferase [41]. A biological explanation for the association between LNCB with health outcomes is inconclusive. LNCB has been suggested to disturb reward systems, activate the cephalic phase insulin response, and induce gut microbiota dysbiosis, which can further lead to insulin resistance and the development of type 2 diabetes [48, 49]. However, more studies including experimental studies in humans as well as long-term evaluations in large cohorts are still needed to further investigate these hypotheses [7, 50].

To date, only a few studies specifically explored associations of FJ with health outcomes. Our results suggest an inverse association when consuming ≤2 servings/day compared to no intake, which has also been observed in a dose–response meta-analysis of two cohorts investigating the association between FJ intake (75–150 ml/day) and MetS incidence [15]. Khan et al. also demonstrated an inverse association between FJ intake of ~150 ml and CVD incidence, but not at higher intake levels [51]. A recent and extensive meta-analysis by D’Elia also suggested a non-linear association between low to moderate 100% FJ intake (<80 ml/day) with incidence rates of stroke and CVD [17]. This inverse association among participants with moderate intake levels might be due to overall healthier behaviours of participants within this intake group. However, this notion is not clearly seen in our study populations as the distribution of characteristics was comparable across groups in all studies. Possible underlying mechanisms of the inverse associations between moderate intake of FJ and NAFLD include the beneficial composition of FJ, which may contain a certain amount of antioxidants (i.e polyphenols) and other bioactive components (i.e vitamins, fibre and minerals) that enhance metabolic profile [15, 17, 51, 52]. However, its high caloric content and the presence of fructose may be counterproductive to the benefits of consuming higher quantities of FJ.

Strengths of our study include the large sample size derived from several European population-based studies, the opportunity to control for a wide range of relevant confounders and to perform stratified analysis particularly in Lifelines which comprised the largest cohort. We conducted the harmonized statistical analyses across cohorts including substitution analyses which to date, are often lacking in recent studies. This is the first meta-analysis focusing on NAFLD assessed by FLI, which facilitated non-invasive and simple assessment in larger studies. There are also several limitations to this study. Dietary intake was self-reported, hence bias due to social desirability cannot be excluded. Although the FFQs used in current study were extensively evaluated for a range of nutrients and foods, they were not specifically designed to examine SSB, LNCB, and specific sweeteners intakes. Moreover, SSB, LNCB, and FJ intakes in our study were relatively low, which may explain the lower risk estimates in our study when compared to some previous ones i.e from the US [6, 7, 13]. SSB intake in our Dutch study populations was lower than that reported in the Dutch National Food Consumption Survey, which may be because SSB was grouped together with carbonated/soft/isotonic drinks and diluted syrups. We used FLI as a proxy for NAFLD, which has limitations including its inability to distinguish moderate to severe steatosis from mild steatosis [9, 53]. The gold standard for NAFLD diagnosis is liver biopsy which is an invasive and costly procedure. To avoid these consequences, non-invasive imaging procedures have been introduced, i.e ultrasound or magnetic resonance. However, it also has limitations associated with availability and cost for large-scale studies, therefore use of serum biomarkers is preferred. FLI has been externally validated in several European and Asian populations [10, 54–57], and has been endorsed to be the best-validated tool to detect the presence of steatosis [58, 59]. Despite adjustment for a wide range of confounders, residual confounding may still be present. However, additional sensitivity analysis using the *E*-value [60] showed that any unmeasured confounder would need to be quite strongly associated with both exposure and outcome to explain away the association (data not shown), which is not very likely based on the results for the confounders included. Finally, as always in a cross-sectional study, reverse causality cannot be excluded.

In conclusion, we observed adverse associations between SSB and LNCB intakes and FLI-defined NAFLD prevalence, as well as between replacement of SSB with the same amount of LNCB and FLI-defined NAFLD, which may partly be explained by reverse causality. Our findings suggest a beneficial association between moderate intake of FJ and FLI-defined NAFLD at intake level of ≤2 servings/day when compared to no intake. These findings provide additional information on the potential adverse impact of sweetened beverages on health. Longer-term prospective studies with objective methods determining the intake of sugar and sweeteners are warranted to further substantiate our findings.

## Supplementary information

SUPPLEMENTAL MATERIAL

Supplemental Table 2

Supplemental Table 3

Supplemental Table 4

Supplemental Table 5

Supplemental Table 6

Supplemental Table 7

## Data Availability

The datasets generated during and/or analysed during the current study are available from the corresponding author on reasonable request and after approval from the relevant partners.
