# Supplementary material for: Sugar-sweetened beverages, low/no-calorie beverages, fruit juice and non-alcoholic fatty liver disease defined by fatty liver index: the SWEET project
Source: Nutr Diabetes. 2023 Apr 21;13:6. doi: 10.1038/s41387-023-00237-3 (PMC10121594; doi:10.1038/s41387-023-00237-3)
Supplement: Supplementary file 1 — SUPPLEMENTAL MATERIAL [file 41387_2023_237_MOESM1_ESM.docx]

**Supplemental Table 1** Flow chart of participants’ inclusion for all cohorts

|  | **Lifelines** | **NQPlus^a^** | **PREDIMED-plus** | **AOC** |
| --- | --- | --- | --- | --- |
| N total participants | 152278 men and women aged ≥18y | 2048 men and women aged 20-70 y | 266 men aged 55-75y and women aged 60-75y with metabolic syndrome | 4837 Dutch patients aged 60-80y diagnosed with MI within 10y prior to study enrolment |
| *Exclusion* |  |  |  |  |
| - Missing dietary intake | N excluded = 8633 > N = 144095 | N excluded = 401 > N = 1647 | N excluded = 0 > N = 266 | N excluded = 453 > N = 4384 |
| - Implausible energy intake (men with energy intake <800 or >4000 kcal/d or women with energy intake <500 or >3500 kcal/d) | N excluded = 15,483 > N = 128612 | N excluded = 20 > N = 1627 | N excluded = 3 > N = 263 | N excluded = 24 > N = 4360 |
| - Missing outcome | N excluded = 86137 > N = 42475 | N excluded = 56 > N = 1361 | N excluded = 10 > N = 253 | N excluded = 179 > N = 4181 |
| - History of hepatitis | N excluded = 31 > N = 42444 | N excluded = 32 > N = 1525 | NA | NA |
| - Excessive alcohol consumption(>20 gram/d for women or >30 gram/d for man) | N excluded = 1825 > N = 40619 | N excluded = 184 > N = 1341 | N excluded = 1 > N = 252 | N excluded = 647 > N = 3534 |
| - Missing covariates |  |  |  |  |
| - - Physical activity | N excluded = 3318 (8%) > N = 37301 | N excluded = 110 (8%^b^) | N missing = 0 | N missing = 22 (<1%) > N = 3512 |
| - - Sedentary activity | N missing = 0 | N excluded = 111 (8%^b^) | N missing = 0 | NA |
| - - Education level | N excluded = 216 (<1%) > N = 37085 | N missing = 0 | N excluded = 2 (<1%) > N = 250 | N missing = 19 (<1%) > N = 3493 |
| - - Smoking status | N excluded = 145 (<1%) > N = 36940 | N missing = 126 (9%^b^) | N missing = 0 | N missing = 0 |
| N for analysis | 36940 | 1341 | 250 | 3493 |

^a^ multiple imputation was performed for missing covariates

^b^ of the total participants.

Abbreviation: MI, myocardial infarction.
